# Supplementary material for: Factors Associated with High Mercury Levels in Women and Girls from The Mojana Region, Colombia, 2013–2015
Source: Int J Environ Res Public Health. 2020 Mar 11;17(6):1827. doi: 10.3390/ijerph17061827 (PMC7143275; doi:10.3390/ijerph17061827)
Supplement: Supplementary file 1 [file ijerph-17-01827-s001.pdf]

Table S1. Characteristics of women participants by department.

Variable	Department										
	All		Antioquia		Bolivar		Cordoba		Sucre		*P*-value
**Age Group**	**x̄ or %**	**95% CI**	**x̄ or %**	**95% CI**	**x̄ or %**	**95% CI**	**x̄ or %**	**95% CI**	**x̄ or %**	**95% CI**	
<18	12.4	9.6–15.9	11.3	5.5–17.1	8.8	3.5–14	15.4	8.8–21.9	14.6	6.9–22.4	
18–29	21.5	17.8–25.6	22.6	14.9–30.3	21.1	13.5–28.6	23.9	16.1–31.7	17.1	8.9–25.3	
30–49	40.9	36.3–45.6	45.2	36.1–54.4	45.6	36.4–54.8	32.5	23.9–41.0	40.2	29.5–50.9	0.356
50–59	16.1	12.9–19.9	10.4	4.8–16.1	14.0	7.6–20.5	21.4	13.9–28.8	19.5	10.8–28.2	
≥60	9.1	6.7–12.2	10.4	4.8–16.1	10.5	4.8–16.2	6.8	2.2–11.4	8.5	2.4–14.6	
**Biological Matrices**											
Mercury in blood µg/L*	3.3	1.6–7.6	4.1	2.2–7.9	1.6	0–4.1	5.4	3.1–11.2	2.9	1.6–7.5	<0.001
Urine mercury µg/L*	6.0	4.3–11.1	9.3	5.2–32	6.9	4.3–9.9	5.2	3.8–9.5	5.2	3.6–6.5	<0.001
Mercury in hair µg/g*	1.2	0.7–2.2	1.0	0.6–1.7	1.1	0.6–1.6	1.7	1.0–2.7	1.2	0.7–2.2	<0.001
**Education**											
Primary	41.7	37.1–46.4	36.5	27.6–45.4	38.9	29.9–47.9	45.3	36.2–54.4	47.6	36.6–58.5	
High school	29.7	25.6–34.2	29.6	21.2–37.9	28.3	19.9–36.7	26.5	18.4–34.5	36.6	26.1–47.1	0.034
Technical /University/ Postgraduate	27.8	23.8–32.3	33.9	25.2–42.6	30.1	21.6–38.6	28.2	19.9–36.4	15.8	7.9–23.8	
**Occupation**											
General services	8.2	5.6–11.7	10.4	4.8–16.1			8.7	3.5–13.9	4.9	0.2–9.6	
Administrative	9.4	6.7–13.2	15.6	8.9–22.3			4.3	0.6–8.1	8.5	2.4–14.6	
Business	4.7	2.8–7.7	6.1	1.7–10.5	NA			NA	9.7	3.2–16.2	<0.001
Nursing Assistant	9.4	6.7–13.2	8.7	3.5–13.9			15.6	8.9–22.3	2.4	0.9–5.8	
Homemaker	40.3	34.9–45.8	31.3	22.7–39.8			44.3	35.2–53.5	50.0	39.2–60.9	
Others	16.7	12.9–21.1	16.5	10.7–23.4			16.5	10.7–23.4	18.3	9.8–26.7	
**Consumption Habits**											
Alcohol	40.7	36.1–45.4	39.1	30.1–48.1	53.2	43.8–62.5	35.9	27.1–44.6	32.9	22.7–43.2	0.082
Cigarette	5.4	3.6–8.0	5.2	1.1–9.3	8.1	2.9–13.2	6.9	1.9–11.9		NA	0.034
Vitamins	46.9	42.2–51.7	37.4	28.5–46.3	54.4	45.2–63.6	43.6	34.5–52.6	54.9	44.0–65.7	0.025
**Background**											
Presence of at least one disease	18.9	15.5–22.9	21.7	14.1–29.3	22.8	15.0–30.6	14.5	8.1–20.9	15.8	7.9–23.8	0.297
**Source of Water Consumption**											
Tap	41.1	36.5–45.9	75.7	67.8–83.6	22.8	15.0–30.6	23.9	16.1–31.7	42.7	31.9–53.5	
Large bottle	6.8	4.7–9.6	14.8	8.2–21.3	1.7	0.7–4.2	5.1	1.1–9.2	4.9	0.2–9.6	
River	15.9	12.7–19.7	2.6	0.3–5.5	23.7	15.8–31.5	6.8	2.2–11.4	36.6	26.1–47.1	<0.001
Well	30.1	25.9–34.7	6.1	1.7–10.5	34.2	25.4–42.9	64.1	55.3–72.9	9.8	3.3–16.2	
Other	6.0	4.1–8.8	0.9	0.8–2.6	17.5	10.5–24.6		NA	6.1	0.9–11.3	
**Fish Consumption Frequency**											
Never	4.4	2.8–6.9	10.4	4.8–16.1	0.9	0.8–2.6	3.4	0.1–6.7	2.5	0.9–5.9	<0.001
Once/month	32.5	28.2–37.2	49.6	40.4–58.8	28.1	19.8–36.4	35.9	27.1–44.7	9.9	3.3–16.4	

Once/week	27.2	23.1–31.6	24.3	16.4–31.2	27.2	18.9–35.4	24.8	16.9–32.7	34.6	24.1–45.0	
2–4 times/week	27.6	23.6–32.1	13.9	7.5–20.3	35.1	26.3–43.9	25.6	17.7–33.6	39.5	28.8–50.2	
Daily	8.2	5.9–11.2	1.7	0.7–4.1	8.8	3.5–14.0	10.3	4.7–15.8	13.6	6.0–21.1	
**Prevalence of High Mercury Values (in any matrix)**	62.8	58.1–67.3	57.4	48.3–64.5	55.3	46.1–64.4	77.8	70.2–85.4	59.8	49.0–70.5	0.001
**Symptoms**											
Metallic Taste in the Mouth	15.6	13.3–20.4	17.4	10.4–24.4	4.4	0.6–8.2	21.4	13.9–28.8	25.6	16.1–35.1	<0.001
Salivation	21.7	18.1–25.9	16.5	9.7–23.4	14.0	7.6–20.5	24.8	16.9–32.7	35.4	24.9–45.8	0.001
Loss of Appetite	39.9	35.4–44.7	33.0	24.4–41.7	39.5	30.4–48.5	39.3	30.4–48.2	51.2	40.3–62.1	0.084
Weight Loss	31.1	26.8–35.6	25.2	17.2–32.2	38.6	29.6–47.6	35.9	27.1–44.7	21.9	12.9–30.9	0.025
Hair Loss	49.3	44.6–54.0	30.4	21.9–38.9	45.6	36.4–54.8	64.9	56.2–73.7	58.5	47.8–69.3	<0.001
Tremors	33.9	29.5–38.5	34.8	26.0–43.6	36.8	27.9–45.8	25.6	17.7–33.6	40.2	29.5–50.9	0.138
Insomnia	49.8	45.0–54.5	66.1	57.4–74.8	30.7	22.2–39.2	36.8	27.9–45.6	71.9	62.1–81.8	<0.001
Nervousness	37.6	33.1–42.3	33.9	25.2–42.6	32.5	23.8–41.1	43.6	34.5–52.6	41.5	30.7–52.2	0.235
Depression	32.9	28.6–37.5	11.3	5.5–17.1	47.4	38.1–56.6	44.4	35.4–53.4	26.8	17.2–36.5	<0.001
Palpitations	46.0	41.3–50.8	31.3	22.8–39.8	55.3	46.1–64.5	47.0	37.9–56.1	52.4	41.5–63.3	0.002

Proportion is presented for qualitative variables and mean for quantitative variables. In some cases, *median and Interquartile Range (IR) are presented, owing to the asymmetric distribution of the variable. NA: no observations.

Table S2. Concentration of mercury levels in the evaluated matrices, grouped according to type of exposure.

Variable	Occupational						Environmental					
	Blood		Urine		Hair		Blood		Urine		Hair	
	Median	IR	Median	IR	Median	IR	Median	IR	Median	IR	Median	IR
**All**	6.9	2.1-13.6	14.0	5.2–26.5	2.3	1.2-3.2	3.1	1.5–7.3	5.6	4.1–9.6	1.2	0.6–2.1
**Age group**												
<18			NE				2.3	1.4–4.5	9.3	5.1 – 15.0	1.4	0.8–2.2
18–29	7.1	0–27.9	17	14.0–32.0	1.6	0.9–2.9	2.8	1.6–6.3	5.6	3.8–10.7	1.1	0.7–1.7
30–49	4.3	2.1–8.1	12.3	4.7–24.2	1.7	1.2–3.6	3.3	1.6–6.6	5.7	3.8– 8.9	1.2	0.6–2.1
50–59	11.8	5.7–23.2	8.5	5.2–14.0	2.8	2.3–3.0	4.5	1.6–9.4	5.0	4.2– 6.2	1.3	0.7–2.6
≥ 60			NE				2.9	1.0–5.6	6.0	3.8–10.0	1.0	0.5–1.8
**Enrollment in the government health system**												
Contributive	10.3	6.9–23.2	22	14.0–32.0	1.9	1.5–3.6	3.4	1.9–6.4	6.6	4.0–10.0	0.9	0.6–1.4
Subsidized	4.3	1.3–7.6	5.3	4.5–17.0	2.3	1.1–3.2	2.9	1.5–7.1	5.5	4.1–8.8	1.2	0.7–2.3
No-affiliation			NE		1.7	0.7–2.8	11.9	4.1–28.5	6.8	4.5–9.6	2.2	1.5–7.4
**Department**												
Bolivar	0	0–1.3	8.0	3.9–22.0	1.2	0.7–1.5	1.7	0–4.2	6.2	4.7–9.7	1.1	0.6–1.9
Sucre	2.7	0.7–4.3	5.3	2.7–17.0	1.1	0.8–2.5	2.9	1.6–8.6	5.1	3.6–6.5	1.2	0.7–2.2

Antioquia	15.3	8.3–33.3	25.7	5.4–52.7	3.0	1.6–3.6	3.1	2.0–6.7	8.8	4.8–27.0	0.9	0.5–1.5
Cordoba	7.3	4.9–11.8	14	7.0–15.0	2.4	1.7–3.2	5	2.8–10.8	4.5	3.6–6.9	1.6	0.9–2.6
**Education**												
Primary	6.9	2.6–9.8	7.6	4.7–22.0	1.9	1.2–3.2	4.1	1.6–10.2	5.3	3.8–7.7	1.4	0.7–2.5
High school	6.3	0.7–27.9	19.5	6.1–52.7	2.5	1.1–3.0	2.5	1.4–5.7	8.6	4.8–15.0	1.2	0.7–1.9
Technical/University/ Postgraduate	8.1	1.0–10.3	14.0	8.0–14.0	2.0	1.5–3.0	3.0	1.7–5.3	5.6	3.6–10.0	0.9	0.6–1.4
**Symptoms**												
Metallic taste in the Mouth	4.7	2.1–7.6	14.0	5.0–22.0	1.7	1.2–2.4	4.8	1.9–9.2	4.5	2.8–5.9	1.4	0.8–2.8
Salivation	7.1	4.2–12.1	14.5	5.0–25.0	2.3	1.5–2.5	3.6	1.6–7.7	4.6	3.2–6.0	1.5	0.9–2.5
Loss of Appetite	4.1	1.3–6.9	8.0	4.5–25.0	1.5	0.8–2.5	2.7	1.5–6.9	4.9	3.7–6.5	1.2	0.7–2.2
Weight Loss	2.1	0.7–12.1	15.0	5.0–22.0	1.3	0.8–2.3	3.1	1.5–8.2	5.6	4.3–8.9	1.3	0.7–2.6
Hair Loss	4.3	1.3–8.1	12.0	5.3–23.5	1.6	1.1–2.8	3.1	1.5–7.9	5.3	3.7–8.3	1.2	0.7–2.1
Tremors	6.9	1.3–15.3	14.5	5.0–25.0	1.5	1.2–2.5	2.4	1.3–8.1	5.6	4.0–9.8	1.2	0.8–2.4
Insomnia	6.9	2.1–15.3	10.0	5.0–32.0	2.5	1.5–3.2	3.1	1.7–7.3	5.6	4.2–10.0	1.1	0.7–1.9
Headache	5.7	1.3–8.2	10.0	5.0–22.0	1.7	1.2–2.9	3.0	1.5–7.5	5.5	3.6–8.3	1.2	0.7–2.2
Palpitations	3.3	0–8.2	14.0	5.0–22.0	1.7	1.1–2.5	2.9	1.4–7.6	5.4	3.9–8.6	1.2	0.7–2.4

IR: interquartile range, NE: non-estimable due to low number of observations.

Table S3. Logistic regression models for high levels of mercury in all biological matrices and for each matrix.

Covariate	OR	95% CI	*P*-value
**High Mercury in any of the Matrices**			
Age			
>18		Reference	
18–29	0.7	0.2–2.6	0.607
30–49	0.4	0.1–1.7	0.249
50–59	0.9	0.2–3.8	0.932
≥60	0.3	0.1–1.4	0.127
Water source			
Tap		Reference	
Large bottle	1.8	0.7–4.6	0.200
River	1.7	0.7–4.1	0.208
Well	3.6	1.9–6.9	<0.001
Others	2.1	0.3–13.2	0.433
Education			
Primary		Reference	
High school	0.8	0.4–1.7	0.665
Technical/University	1.0	0.4–2.6	0.960
Fish consumption frequency			
Never		Reference	
Once/month	3.7	1.1–12.8	0.034
Once/week	4.5	1.3–15.7	0.017
2–4 times/week	6.9	1.9–24.6	0.003
Diary	16.6	3.0–90.9	0.001
Occupation			
General services		Reference	
Administrative	0.7	0.2–2.7	0.639
Business	1.2	0.3–5.7	0.789
Homemaker	0.9	0.3–2.7	0.934
Nursing assistant	0.2	0.1–0.9	0.040
Others	0.3	0.1–1.2	0.092
**High mercury in blood***			
Fish consumption frequency			
Never		Reference	
Once/month	1.6	0.3–9.8	0.559
Once/week	1.4	0.2–8.1	0.724
2–4 times/week	2.4	0.4–14.0	0.349
Daily	3.8	0.6–26.3	0.175
**High mercury in urine***			
Fish consumption frequency			
Never		Reference	
Once/month	0.4	0.0–3.4	0.427
Once/week	0.3	0.0–2.3	0.258
2–4 times/week	0.2	0.0–2.0	0.177
Daily		NE	
**High mercury in hair***			
Fish consumption frequency			
Never		Reference	
Once/month	5.5	1.3–22.7	0.019
Once/week	7.9	1.9–33.2	0.005
2–4 times/week	11.2	2.6–48.2	0.001
Daily	33.7	5.3–215.6	< 0.001

*Adjusted for age, source of drinking water, education, and occupation. NE: not estimated, OR: Odds Ratio estimation.
